# Streaming Quantiles Algorithms with Small Space and Update Time

**DOI:** 10.3390/s22249612

**Published:** 2022-12-08

**Authors:** Nikita Ivkin, Edo Liberty, Kevin Lang, Zohar Karnin, Vladimir Braverman

**Affiliations:** 1 Amazon, New York, NY 10001, USA; 2 Pinecone, San Mateo, CA 94402, USA; 3 Yahoo Research, Sunnyvale, CA 94089, USA; 4 Department of Computer Science, Rice University, Houston, TX 77005, USA

**Keywords:** sketching, quantiles, streaming

## Abstract

Approximating quantiles and distributions over streaming data has been studied for roughly two decades now. Recently, Karnin, Lang, and Liberty proposed the first asymptotically optimal algorithm for doing so. This manuscript complements their theoretical result by providing a practical variants of their algorithm with improved constants. For a given sketch size, our techniques provably reduce the upper bound on the sketch error by a factor of two. These improvements are verified experimentally. Our modified quantile sketch improves the latency as well by reducing the worst-case update time from $O(\frac{1}{\varepsilon })$ down to $O(\log\frac{1}{\varepsilon })$.

## 1. Introduction

Estimating the underlying distribution of data is crucial for many applications. It is common to approximate an entire Cumulative Distribution Function (CDF) or specific quantiles. The median (0.5 quantile) and 95-th and 99-th percentiles are widely used in financial metrics, statistical tests, and system monitoring. Quantiles summary found applications in databases [1,2], sensor networks [3], logging systems [4], distributed systems [5], and decision trees [6]. While computing quantiles is conceptually very simple, doing so naively becomes infeasible for very large data.

Formally, the quantiles problem can be defined as follows. Let *S* be a multiset of items $S={\left\{s_{i}\right\}}_{i=1}^{n}$. The items in *S* exhibit a full-ordering and the corresponding smaller-than comparator is known. The rank of a query *q* (with regard to *S*) is the number of items in *S*, which are smaller than *q*. An algorithm should process *S* such that it can compute the rank of any query item. Answering rank queries exactly for every query is trivially possible by storing the multiset *S*. Storing *S* in its entirety is also necessary for this task. An approximate version of the problem relaxes this requirement. It is allowed to output an approximate rank, which is off by at most εn from the exact rank. In a randomized setting, the algorithm is allowed to fail with probability at most δ. Note that, for the randomized version to provide a correct answer to all possible queries, it suffices to amplify the success probability by running the algorithm with a failure probability of δε, and applying the union bound over $O(\frac{1}{\varepsilon })$ quantiles. Uniform random sampling of $O(\frac{1}{\varepsilon^{2}}\log\frac{1}{\delta \varepsilon })$ solves this problem.

In network monitoring [7] and other applications, it is critical to maintain statistics while making only a single pass over the data and minimizing the communication and update time. As a result, the problem of approximating quantiles was considered in several models, including distributed settings [5,8,9], continuous monitoring [10,11], streaming [12,13,14,15,16,17,18], and sliding windows [19,20]. In the present paper, the quantiles problem is considered in a standard streaming setting. The algorithm receives the items in *S* one by one in an iterative manner. The algorithm’s approximation guarantees should not depend on the order or the content of the updates $s_{t}$, and its space complexity should depend on *n* at most poly-logarithmically (Throughout this manuscript, we assume that each item in the stream requires O(1) space to store).

In their pioneering paper [21], Munro and Paterson showed that one would need $\Omega (n^{1/p})$ space and *p* passes over the dataset to find a median. They also suggested an optimal iterative algorithm to find it. Later Manku et al. [14] showed that the first iteration of the algorithm in [21] can be used to solve the ε approximate quantile problem in one pass using only $O(\frac{1}{\varepsilon }\log^{2}n)$ space. Note that, for a small enough ε, this is a significant improvement over the naive algorithm, which samples $O(\frac{1}{\varepsilon^{2}}\log\frac{1}{\varepsilon })$ items of the stream using reservoir sampling. The algorithm in [14] is deterministic; however, compared with reservoir sampling, it assumes the length of the stream is known in advance. In many applications, such an assumption is unrealistic. In their follow-up paper [15], the authors suggested a randomized algorithm without that assumption. Further improvement by Agarwal et al. [12] via randomizing the core subroutine pushed the space requirements down to $O(\frac{1}{\varepsilon }\log^{3/2}\frac{1}{\varepsilon })$. Additionally, the new data structure was proven to be fully mergeable. Greenwald and Khanna in [17] presented an algorithm that maintains upper and lower bounds for each quantile individually, rather than one bound for all quantiles. It is deterministic and requires only $O(\frac{1}{\varepsilon }\log\varepsilon n)$ space. It is not known to be fully mergeable. Later, Felber and Ostrovsky [22] suggested non-trivial techniques of feeding sampled items into sketches from [17] and improved the space complexity to $O(\frac{1}{\varepsilon }\log\frac{1}{\varepsilon })$. Recently, ref. [13] presented an asymptotically optimal but non-mergeable data structure with space usage of $O(\frac{1}{\varepsilon }\log\log\frac{1}{\varepsilon })$ and a matching lower bound. They also presented a fully mergeable algorithm whose space complexity is $O(\frac{1}{\varepsilon }\log^{2}\log\frac{1}{\varepsilon })$.

In the current paper, we suggest several further improvements to the algorithms introduced in [13]. These improvements do not affect the asymptotic guarantees of [13], but reduce the upper bounds by constant terms, both in theory and practice. The suggested techniques also improve the worst-case update time. All the algorithms presented operate in the comparison model. They can only store (and discard) items from the stream and compare between them, and can not “compute” using averaging or going over entire dictionary. For example list of strings of unbounded length can not be enumerated, thus if some item was “forgotten”, it cannot be returned as an output. For this reason, we keep aside a line of work finding quantiles under a fixed dictionary: [23,24,25] and others. For more background on quantile algorithms in the streaming model, see [16,18].

## 2. A Unified View of Randomized Solutions

**Figure 1:**
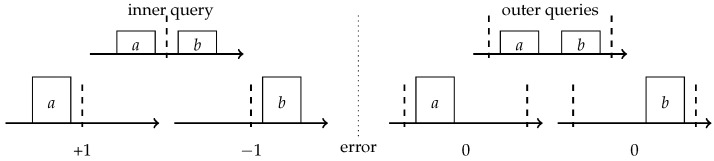
One pair compression for (*a*,*b*) introduces ±1 rank error to inner queries and no error to outer queries.

To introduce further improvements to the streaming quantiles algorithms, we will first re-explain the previous work using simplified concepts of one pair compression and a compactor. Consider a simple problem in which your data set contains only two items *a* and *b*, while your data structure can only store one item. We focus on the comparison-based framework where we can only compare items and cannot compute new items via operations such as averaging. In this framework, the only option for the data structure is to pick one of them and store it explicitly. The stored item *x* is assigned weight 2. Given a rank query *q* the data structure will report 0 for q<x, and 2 for q>x. For q∉[a,b] the output of the data structure will be correct; however, for q∈[a,b], the correct rank is 1 and the data structure will output with 0 or 2. It, therefore, introduces a +1/−1 error depending on which item was retained. From this point on, *q* is an inner query with respect to the pair (a,b) if q∈[a,b] and an outer query otherwise. This lets us distinguish those queries for which an error is introduced from those that were not influenced by a compression. Figure 1 depicts the above example of one pair compression.

The example gives rise to a high-level method for the original problem with a dataset of size *n* and memory capacity of *k* items. Namely, (1) keep adding items to the data structure until it is full; and (2) choose any pair of items with the same weight and compress them. Notice that if we choose those pairs without care, in the worst case, we might end up representing the full dataset by its top *k* elements, introducing an error of almost *n*, which is much larger than εn. Intuitively, pairs being compacted (compressed) should close ranks, thereby affecting as few queries as possible.

**Figure 2:**
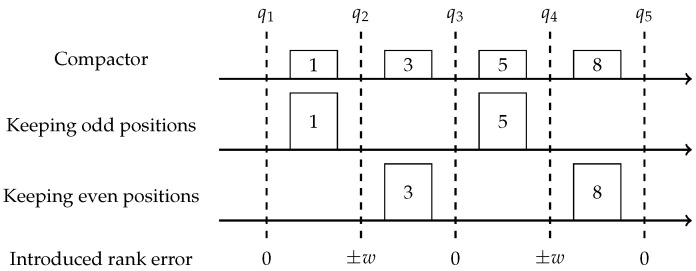
Compacting [1,3,5,8] introduces ±w rank error to inner queries $q_{2,4}$ and no error to outer queries $q_{1,3,5}$.

This intuition is implemented via a compactor. First introduced by Manku et al. in [14], it defines an array of *k* items with weight *w* each, and a compaction procedure which compress all *k* items into k/2 items with weight 2w. A compaction procedure first sorts all items, then deletes either even or odd positions and doubles the weight of the rest. Figure 2 depicts the error introduced for different rank queries *q*, by a compaction procedure applied to an example array of items [1,3,5,8]. Notice that the compactor utilizes the same idea as the one pair compression, but on the pairs of neighbors in the sorted array; thus by performing k/2 non-intersecting compressions, it introduces an overall error of *w* as opposed to kw/2.

The algorithm introduced in [14], defines a stack of $H=O(\log\frac{n}{k})$ compactors, each of size *k*. Each compactor obtains as an input a stream and outputs a stream with half the size by performing a compact operation each time its buffer is full. The output of the final compactor is a stream of length *k* that can simply be stored in memory. The bottom compactor that observes items has a weight of 1; the next one observes items of weight 2 and the top one $2^{H-1}$. The output of a compactor on *h*-th level is an input of the compactor on (h+1)-th level. Note that the error introduced on *h*-th level is equal to the number of compactions $m_{h}=\frac{n}{kw_{h}}$ times the error introduced by one compaction $w_{h}$. The total error can be computed as: $\mathrm{Err}=\sum_{h=1}^{H}m_{h}w_{h}=H\frac{n}{k}=O\left(\frac{n}{k}\log\frac{n}{k}\right).$ Setting $k=O(\frac{1}{\varepsilon }\log\varepsilon n)$ will lead to an approximation error of εn. The space used by *H* compactors of size *k* each is $O(\frac{1}{\varepsilon }\log^{2}\varepsilon n)$. Note that the algorithm is deterministic.

Later, Agarwal et al. [12] suggested the compactor to choose the odd or even positions randomly and equiprobably, pushing the introduced error to zero in expectation. Additionally, the authors suggested a new way of feeding a subsampled streams into the data structure, recalling that $O(\frac{1}{\varepsilon^{2}}\log\frac{1}{\varepsilon })$ samples preserve quantiles with ±εn approximation error. The proposed algorithm requires $O(\frac{1}{\varepsilon }\log^{3/2}\frac{1}{\varepsilon })$ space and succeeds with high constant probability.

To prove the result the authors introduced a random variable $X_{i,h}$ denoting the error introduced on the *i*-th compaction at *h*-th level. Then, the overall error is $\mathrm{Err}=\sum_{h=1}^{H}\sum_{i=1}^{m_{h}}w_{h}X_{i,h},$ where $w_{h}X_{i,h}$ is bounded, has mean zero and is independent of the other variables. Thus, due to the Hoeffding’s inequality:

$$P\left(|\mathrm{Err}|>\varepsilon n\right)\leq 2\exp\frac{-\varepsilon^{2}n^{2}}{\sum_{h=1}^{H}\sum_{i=1}^{m_{h}}w_{h}^{2}}.$$

Setting $w_{h}=2^{h-1}$ and $k=O\left(\frac{1}{\varepsilon }\sqrt{1/(\varepsilon \delta )}\right)$ will keep the error probability bounded by δ for $O\left(\frac{1}{\varepsilon }\right)$ quantiles.

The following improvements were made by Karnin et al. [13].

1. Use exponentially decreasing size of the compactor. Higher weighted items receive higher capacity compactors.
2. Replace compactors of capacity 2 with a sampler. This retains only the top $O(\log\frac{1}{\varepsilon })$ top compactors.
3. Keep the size of the top O(loglog1/δ) compactors fixed.
4. Replace the top O(loglog1/δ) compactors with a GK sketch [17].

(1) and (2) reduced the space complexity to $O(\frac{1}{\varepsilon }\sqrt{\log1/\varepsilon })$, (3) pushed it further to $O(\frac{1}{\varepsilon }\log^{2}\log\frac{1}{\varepsilon })$, and (4) led to an optimal $O(\frac{1}{\varepsilon }\log\log\frac{1}{\varepsilon }).$ The authors also provided a matching lower bound. Note, the last solution is not mergeable due to the use of GK [17] as a subroutine.

While (3) and (4) lead to the asymptotically better algorithm, its implementation is complicated for application purposes and mostly are of a theoretical interest. In this paper, we build upon the KLL algorithm of [13] using only (1) and (2).

In [13], the authors suggest the size of the compactor to decrease as $k_{h}=c^{H-h}k$, for c∈(0.5,1), then $\sum_{h=1}^{H}\sum_{i=1}^{m_{h}}w_{h}^{2}\leq n^{2}/\left(k^{2}C\right)$ and $P\left(|\mathrm{Err}|>\varepsilon n\right)\leq 2\exp\left(-C\varepsilon^{2}k^{2}\right)\leq \delta ,$ where $C=2c^{2}\left(2c-1\right)$ (In fact [13] has a fixable mistake in their derivation. For the sake of completeness in Appendix A, we clarify that the original results hold, although with slightly different constant terms). Setting $k=O\left(\frac{1}{\varepsilon }\sqrt{\log1/\varepsilon }\right)$ leads to the desired approximation guarantee for all $O\left(1/\varepsilon \right)$ quantiles with constant probability. Note that the smallest meaningful compactor has size 2, thus the algorithm will require $k\left(1+c+\ldots +c^{log_{1/c}k}\right)+O\left(\log n\right)=\frac{k}{1-c}+O\left(\log n\right)$ compactors, where the last term is due to the stack of compactors of size 2. The authors suggested replacing that stack with a basic sampler, which picks one item out of every $2^{w_{H-log_{1/c}k}}$ updates at random and logically is identical, but consumes only O(1) space. The resulting space complexity is $O(\frac{1}{\varepsilon }\sqrt{\log1/\varepsilon })$. We provide the pseudocode for the core routine in Algorithm 1.
**Algorithm 1** Core routines for KLL algorithm [13]  1:**function** KLL.update(item)  2:    **if** Sampler(item) **then** KLL[0].append(item)  3:    **for** h=1…H **do**  4:        **if** len(KLL[*h*] ≥ $k_{h}$) **then** KLL.compact(*h*)  5:    **end for**  6:**end function**  7:**function** KLL.compact(h)  8:    KLL[*h*].sort(); rb = random({0,1});  9:    KLL[h+1].extend(KLL[*h*][rb : : 2])10:    KLL[*h*]= []11:**end function**

## 3. Our Contribution

Although the asymptotic optimum is already achieved for the quantile problem, there remains room for improvement from a practical perspective. In what follows, we provide novel modifications to the existing algorithms that improve both their memory consumption and run-time. In addition to the performance, we ensure the algorithm is easy to use by having the algorithm require only a memory limit, as opposed to versions that must know the values of ε,δ in advance. Finally, we benchmark our algorithm empirically.

### 3.1. Lazy Compactions

Consider a simplified model, when the length of the stream *n* is known in advance. One can easily identify the weight on the top layer of the KLL data structure, as well as the sampling rate and the size of each compactor. Additionally, these parameters do not change while processing the stream. Then, note that while we are processing the first half of the stream, the top layer of KLL will be at most half full, i.e., half of the top compactor memory will not be in use during processing first n/2 items. Let *s* be the total amount of allocated memory and *c* be the compactor size decrease rate. The top layer is of size s(1−c), meaning that a fraction of (1−c)/2 is not used throughout that time period. The suggested value for *c* is $1/\sqrt{2}$, which means that this quantity is 15%. This is of course a lower estimate as the other layers in the algorithm are not utilized in various stages of the processing. A similar problem arises when we do not know the final *n* and keep updating it online: When the top layer is full, the algorithm compacts it into a new layer; at this moment, the algorithm basically doubles its guess of the final *n*. Although after this compaction k/2 items immediately appear on the top layer, we still have 1/4 of the top layer not in use until the next update of *n*. This unused fraction accounts for 7% of the overall allocated memory.

**Figure 3:**
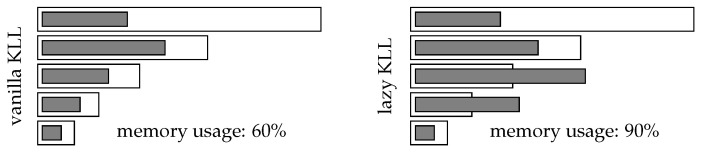
Compactor saturation: vanilla KLL vs. lazy KLL. Empty bar depicts the max capacity of the layer while filled bar depicts utilized capacity of that layer: vanilla KLL ensures that individual layer utilization is never crossing the max capacity of the layer, while lazy KLL ensures only than total utilization across all layers is never higher that total max capacity across all layers.

**Figure 4:**
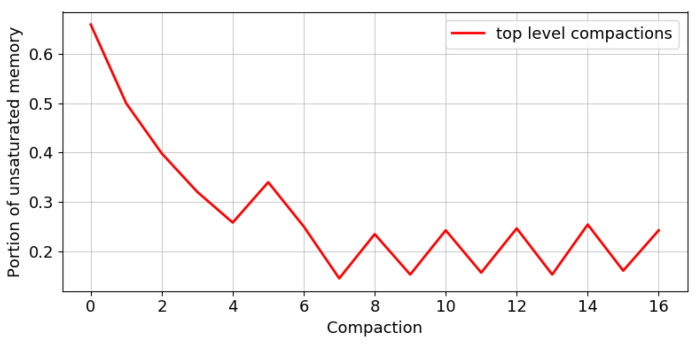
Portion of unsaturated memory when compacting the top layer.

We suggest all the compactors share the pool of allocated memory and perform a compaction only when the pool is fully saturated. This way, each compaction is applied to a potentially larger set of items compared to the fixed budget setting, leading to less compactions. Each compaction introduces a fixed amount of error, thus the total error introduced is lower. Figure 3 visualizes the advantage of using a shared pool of memory. In Figure 4, you can see that the memory is indeed unsaturated even when we compact the top level.

Given the need to have a shared memory comes the question of how to perform the compact operations. The idea is to find the lowest layer that has more items in its buffer than its minimum capacity, and compact it. This scheme is formally defined in Algorithm 2.
**Algorithm 2** Update procedure for lazy KLL  1:**function** KLL.Update(item)  2:    **if** Sampler(item) **then**  3:        KLL[0].append(item);itemsN++;  4:    **end if**  5:    **if** itemsN > sketchSpace **then**  6:        **for** h=1…H **do**  7:           **if** len(KLL[*h*]) ≥$k_{h}$ **then**  8:                KLL.compact(*h*); **break**;  9:           **end if**10:        **end for**11:    **end if**12:**end function**

### 3.2. Reduced Randomness via Anti-Correlations

Consider the process involving a single compactor layer. A convenient way of analyzing its performance is viewing it as a stream processing unit. It receives a stream of size *n* and outputs a stream of size n/2. When collecting *k* items, it sorts them and outputs (to the output stream) either those with even or odd locations. A deterministic compactor may admit an error of up to n/k. A compactor that decides whether to output the even or odds uniformly at random at every step admits an error of $\sqrt{n/k}$ in expectation as the directions of the errors are completely uncorrelated. Here, we suggest a way to force a negative correlation that reduces the mean error by a factor of $\sqrt{2}$. The idea is to group the compaction operations into pairs. At the (2i)-th compaction, choose uniformly at random whether to output the even or odd items, as described above. In the (2i+1)-th compaction, perform the opposite decision compared to the (2i)-th compaction. This way, each coin flip defines 2 consecutive compactions: with probability $\frac{1}{2}$, it is even → odd (e→o), and with probability $\frac{1}{2}$, it is odd → even (o→e).

Let us analyze the error under this strategy. Recall that for a rank query *q* and a compaction operation, *q* is either an inner or outer query. If it is an outer query, it suffers no error. If it is an inner query, it suffers and error of +w if we output the odds and −w if we output evens. Consider the error associated with a single query after two consecutive and anti-correlated compactions. We represent the four possibilities of *q* as io(inner-outer), oi, ii, oo.

Clearly, in expectation, every two compactions introduce 0 error. Additionally, we conclude that instead of suffering an error of up to ±w for every single compaction operation, we suffer that error for every two compaction operations. It follows that the variance of the error is twice smaller, hence the mean error is cut by a factor of $\sqrt{2}$.

### 3.3. Error Spreading

**Figure 5:**
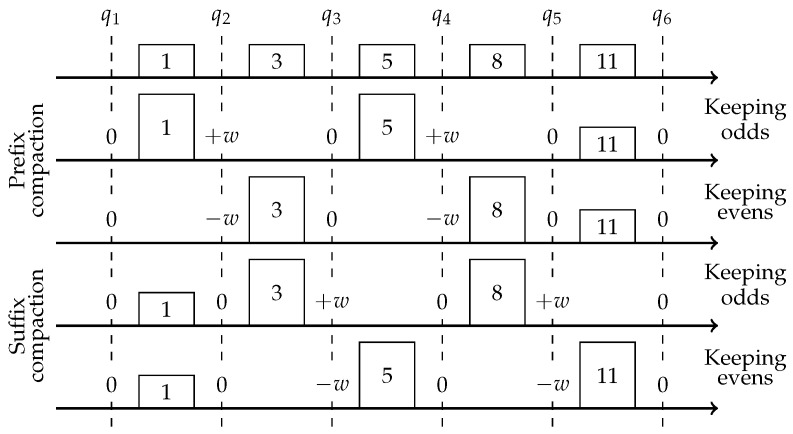
Error analysis for a single query during a compaction. There are now four possibilities: prefix/suffix compaction, keep even/odd positions.

Recall the analysis of all compactor-based solutions [12,13,14,16]. During a single compaction, we can distinguish two types of rank queries: inner queries, for which some errors are introduced, and outer queries, for which no error is introduced. Though the algorithms use this distinction in their analysis, they do not take an action to reduce the number of inner queries. It follows that for an arbitrary stream and an arbitrary query, the query may be an inner query the majority of the time, as it is treated in the analysis. In this section, we provide a method that makes sure that a query has an equal chance of being inner or outer, thereby cutting in half the variance of the error associated with any query, for any stream. Consider a single compactor with a buffer of *k* slots, and suppose *k* is odd. On each compaction, we flip a coin and then either compact the items with indices 1 to k−1 (prefix compaction) or 2 to *k* (suffix compaction) equiprobably. This way each query is either inner or outer equiprobably. Formally, for a fixed rank query *q*: with probability at least $\frac{1}{2}$, it is an outer query and then no error is introduced, with probability at most $\frac{1}{4}$, it is an inner query with error −w; and with probability at most $\frac{1}{4}$, it is an inner with error +w. We thus still have an unbiased estimator for the query’s rank, but the variance is cut in half. We note that the same analysis applies for two consecutive compactions using the reduced randomness improvement discussed earlier: The configuration (ii, io, oi, oo) of a query in two consecutive compactions described in Table 1 will now happen with equal probability, hence we have the same distribution for the error: 0 with probability at least $\frac{1}{2}$, +w and −w with probability at most $\frac{1}{4}$ each, meaning that the variance is cut in half compared to its worse case analysis without the error-spreading improvement. Figure 5 visualizes the analysis of the error for a fixed query during a single compaction operation.

### 3.4. Sweep-Compactor

The error bound for all compactor-based algorithms follows from the property that every batch of k/2 pair compressions is disjoint. In other words, the compactor makes sure that all of the compacted pairs can be partitioned into sets of size exactly k/2, the intervals corresponding to each set are disjoint, and the error bound is a result of this property. In this section, we provide a modified compactor that compacts pairs one at time while maintaining the guarantee that pairs can be split into sets of size at least k/2 such that the intervals corresponding to the pairs of each set are disjoint. Compacting a single pair takes constant time; hence, we reduce the worst-case update time from $O(\frac{1}{\varepsilon })$ to $O(\log\frac{1}{\varepsilon })$. Additionally, for some data streams, the disjoint batch size is strictly larger than k/2, resulting in a reduction in the overall error.

**Figure 6:**
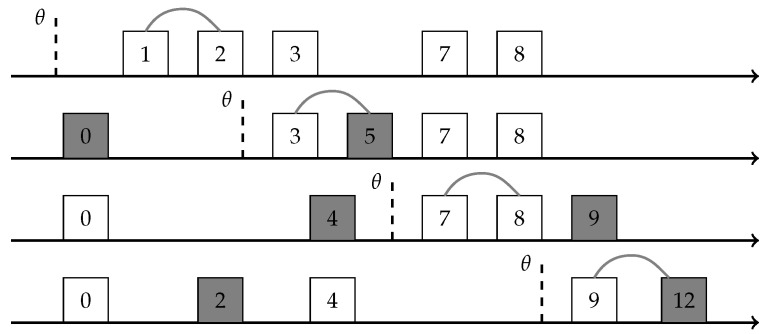
The inner state of a sweep-compactor during a single sweep operation. Notice that in this example, although we have a buffer of size k=5, a single sweep managed to compress 4 pairs, rather than ⌊k/2⌋=2.

The modified compactor operates in phases we call *sweeps*. It maintains the same buffer as before and an additional threshold θ initialized as a special null value. The items in the buffer are stored in non-decreasing sorted order. When we reach capacity, we compact a single pair. If θ is null we set it to −∞ (Notice that −∞ is still defined in the comparison model) or to the value of the smallest item uniformly at random. This mimics the behavior of the prefix/suffix compressions described earlier (If we wish to ignore prefix/suffix compactions, θ should always be initialized to −∞). The pair we compact is a pair of consecutive items where the smaller item is the smallest item in the buffer that is larger than θ (We ignore the case of items with equal value. Note that if that happens, these two items should be compacted together, as this is guaranteed not to incur a loss). If no such pair exists due to θ being too large, we start a new sweep, meaning we set θ to null and act as detailed above. We note that a sweep is the equivalent to a compaction of a standard compactor. Due to this reason, we consistently keep either the smaller or larger item when compacting a single pair throughout a sweep. To keep true to the technique of reduced randomness, we have sweep number 2i+1 draw a coin to determine if the small or large items are kept, and sweep number 2i+2 does the opposite. The pseudo-code for the sweep-compactor is given in Algorithm 3, and Figure 6 visualizes the inner state of the sweep-compactor during a single sweep.
**Algorithm 3** Sweep compaction procedure1:**function** KLLsweep.compact(h)2:    KLL[*h*].sort()3:    $i^{*}={\mathrm{argmin}}_{i}\left(\mathrm{KLL}[h][i]\geq \mathrm{KLL}[h].\theta \right)$4:    **if** $i^{*}==$ **Nonethen** $i^{*}=0$;5:    KLL[h].θ= KLL$\left[i^{*}+1\right]$;6:    KLL[*h*].pop($i^{*}+$ randBit());7:    **return** KLL[*h*].pop($i^{*}$)8:**end function**

Notice that for an already sorted stream, the modified compactor performs only a single sweep, hence in this scenario the resulting error would not be a sum of n/k independent and identically distributed error terms, each of magnitude ±w, but rather a single error term of magnitude ±w. Though this extreme situation may not happen very often, it is likely that the data admits some sorted subsequences and the average sweep would contain more than k/2 pairs. We demonstrate this empirically in our experiments.

## 4. Experimental Results

### 4.1. Data Sets

To study the algorithm properties, we tested it on both synthetic and real datasets, with various sizes, underlying distributions and orders. Note that all the approximation guarantees of the investigated algorithms do not depend on the order in the data; however, in practice, the order might significantly influence the precision of the output within the theoretical guarantees. Surprisingly, the worst-case is achieved when the dataset is randomly shuffled. Therefore, we will pay more attention to randomly ordered data sets in this section. We also experiment with the semi-random orders that resemble more to real life applications. Due to the space limitations, we could not possibly present all the experiments in the paper and present here only the most interesting findings.

Our experiments were carried on the following synthetic datasets: **Sorted** is a stream with all unique items in ascending order. **Shuffled** is a randomly shuffled stream with all unique items. **Trending** is $s_{t}=t/n\;+$ mean-zero random variable. Trending stream mimics a statistical drift over time (widely used in ML). **Brownian** simulates a Brownian motion or a random walk, which generates time series data not unlike CPU usage, stock market, traffic congestion, etc. The length of the stream varies from $10^{5}$ to $10^{9}$ for all the datasets.

In addition to synthetic data, we use two publicly available datasets. The first contains text information and the second contains IP addresses. Both object types have a natural order and can be fed as input to a quantile sketching algorithm.

**(1) Anonymized Internet Traces 2015 (CAIDA)[26]**: The dataset contains anonymized passive traffic traces from the internet data collection monitor, which belongs to CAIDA (Center for Applied Internet Data Analysis) and is located at an Equinix data center in Chicago, IL. For simplicity, we work with the stream of pairs $\left({\mathrm{IP}}_{\mathrm{source}},{\mathrm{IP}}_{\mathrm{destination}}\right)$. The comparison model is lexicographic. We evaluate the performance on the prefixes of the dataset of different sizes: from $10^{7}$ to $10^{9}$. Note that evaluation of the CDF of the underlying distribution for traffic flows helps optimize packet managing. CAIDA’s datasets are used widely for verifying different sketching techniques to maintain different statistics over the flow, and finding quantiles and heavy hitters specifically.
**(2) Page view statistics for Wikimedia projects (Wiki)[27]**: The dataset contains counts for the number of requests for each page of the Wikipedia project during 8 months of 2016. The data is aggregated by day, i.e., within each day data is sorted and each item is assigned with a count of requests during that day. Every update in this dataset is the title of a Wikipedia page. We will experiment with both the original dataset and with its shuffled version. Similarly to CAIDA, we will consider for the Wiki dataset prefixes of size from $10^{7}$ to $10^{9}$. In our experiments, each update is a string containing the name of the page in Wikipedia. The comparison model is lexicographic.

### 4.2. Implementation and Evaluation Details

All the algorithms and experimental settings are implemented in Python 3.6.3. The advantage of using a scripting language is fast prototyping and readable code for distribution inside the community. Time performance of the algorithm is not the subject of the research in the current paper, and we leave its investigation for future work. This in particular applies to the sweep compactor KLL, which theoretically improves the worst-case update time exponentially in $\frac{1}{\varepsilon }$. All the algorithms in the current comparison are randomized, thus for each experiment the results presented are averaged over 50 independent runs. KLL and all suggested modifications are compared with each other and LWYC (the algorithm Random from [28]). In [16], the authors carried on the experimental study of the algorithms from [12,14,15,17] and concluded that their own algorithm (LWYC) is preferable to the others: better in accuracy than [17] and similar in accuracy compared with [15], while LWYC has a simpler logic and easier to implement.

As mentioned earlier, we compared our algorithms under a fixed space restrictions. In other words, in all experiments, we fixed the space allocated to the sketch and evaluated the algorithm based on the best accuracy it can achieve under that space limit. We measured the accuracy as the maximum deviation among all quantile queries, otherwise known as the Kolmogorov–Smirnov divergence, widely used to measure the distance between CDFs of two distributions. Additionally, we measure the introduced variance caused separately by the compaction steps and sampling. Its value can help the user to evaluate the accuracy of the output. Note that for KLL, this value depends on the size of the stream, and is independent of the arrival order of the items. In other words, the guarantees of KLL are the same for all types of streams, adversarial and structured. Some of our improvements change this property; recall that the sweep compactor KLL, when applied to sorted input, requires only a single sweep per layer. For this reason, in our experiments we found variance to be dependent not only on the internal randomness of the algorithm, but also the arrival order of the stream items.

### 4.3. Results

Note that the majority modifications presented in the current paper can be combined for better performance, due to the space limitations we present only some of them. For the sake of simplicity, we will fix the order of suggested modification as: lazy, reduced randomness, error spreading and sweeping, and denote all possible combinations as four 0/1 digits, i.e., 0000 would imply the vanilla KLL without any modifications, while 0011 would imply that we use KLL with error spreading trick and sweeping.

**Figure 7:**
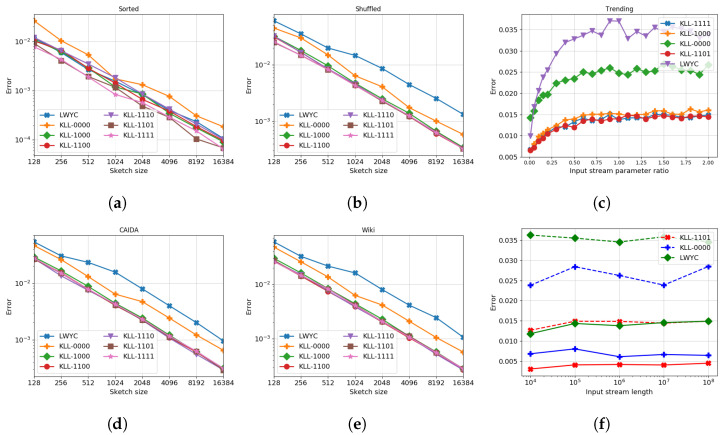

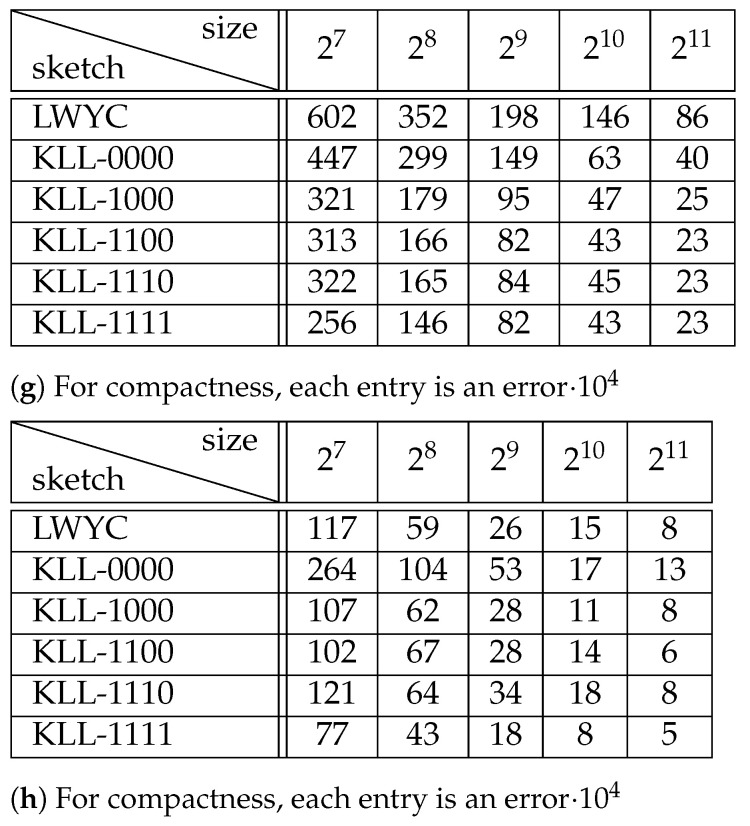
(**a**,**b**,**d**,**e**) depict the trade-off between maximum error over all queried quantiles and the sketch size: (**a**,**b**) test the performance of the algorithms on shuffled and sorted data streams; (**d**,**e**) on CAIDA and Wikipedia datasets correspondingly. (**g**,**h**) show the same trade-off, but make it possible to see the difference between different combos. (**f**) demonstrates independence of the algorithms performance from stream length, dashed lines indicate the sketch size equal 256 and the solid lines correspond to the sketch of size 1024. Finally, (**c**) mix the trending data with a different amounts of a random noise and demonstrates the influence of the stream order on the algorithm precision.

In Figure 7a,b, we compare the size/precision trade-off for LWYC, vanilla KLL, and KLL with modifications. First, we can see that all KLL-based algorithms provide the approximation ratio significantly better than LWYC as the space allocation is growing, which confirms theoretical guarantees. Second, from the experiments, it becomes clear that all algorithms behave worse on the data without any order, i.e., shuffled stream. Although the laziness gives the most significant push to the performance of the Vanilla KLL, all other modifications improve the precision even further if combined. One can easily see it in Figure 7g for the shuffled dataset and Figure 7h for the sorted stream. Same experiments were carried on for the CAIDA dataset (Figure 7d), and shuffled Wikipedia page statistics (Figure 7e).

Although, theoretically, none of the algorithms should depend on the length of the dataset, we verified this property in practice, and the results can be seen on Figure 7f.

In Figure 7c, we verified that although all the theoretical bounds hold, KLL and LWYC performance indeed depends on the amount of randomness in the stream, more randomness leads to less precision. Our experiments were held on the trending dataset, i.e., the stream containing two components: A × (mean-zero random variable) and B × (trend t/n). Figure 7c shows how precision drops as A/B start to grow (X-axis). Note that modified algorithm does not drop in precision as fast as vanilla KLL or LWYC.

## 5. Conclusions

We verified experimentally that the KLL algorithm proposed by Karnin et al. [13] has predicted asymptotic improvement over LWYC [16].We proposed four modifications to KLL with provably better constants in the approximation bounds. Our experiments compared suggested techniques against KLL and LWYC under fixed memory settings: all algorithms obtained the same amount of allocated memory, and we compared the largest deviation from the ground truth among all quantile-queries. Experiments verified that the approximation is roughly twice as good in practice compared to KLL and more than four times better compared to LWYC (and growing with the space allocated to the sketch). Moreover, the worst-case update time for the presented sweep-compactor-based KLL is O(log1/ε), which improves over the rest of the compactor-based algorithms, with vanilla KLL being the next best competitor with an exponentially slower update time of O(1/ε).

## Figures and Tables

**Table 1:** Error of a fixed rank query during two anti-correlated compactions.

	*ii*	*io*	*oi*	*oo*	
even→odd	0	−w	+w	0	w.p. 1/2
odd→even	0	+w	−w	0	w.p. 1/2

## Data Availability

Not applicable.

## Appendix A

### Fixing the Original KLL Proof

The original paper by Karnin et al. [13] contains a mistake regarding the number of compactions performed at a single level. Correcting the mistake is trivial and does not change the authors claim. Nevertheless, we provide a correction of their argument. The authors use compactors of exponentially decreasing size. Higher weight items receive higher capacity compactors. The error appeared in the last inequality of the bound on $m_{h}$—the number of compaction made at level *h* (page 6 in [13]):

(A1) $$m_{h}\leq \frac{n}{k_{h}w_{h}}\leq \frac{2n}{k2^{H}}{\left(2/c\right)}^{H-h}\leq {\left(2/c\right)}^{H-h-1},$$

where *H* is the height of the top compactor, $k_{h}=kc^{H-h}$ is the size of the compactor at height *h*. Note, that the last inequality implies $n\leq ck2^{H-2}=k_{h-1}w_{h-1}$, while from the definition of *H* it follows that at least one compaction happened on level H−1. Therefore, $n\geq k_{h-1}w_{h-1}$. Fixing this slightly increases the constant in the final upper bound.

Recall that k≥4 and c∈(0.5,1). We reuse the notation and refer to the height of the top compactor as *H*. Additionally, we introduce $H^{\prime }$, which denotes the height of the top compactor of size 2. Due to the choice of *k* and *c*, we can conclude that $H^{\prime }\leq H-1$.

Every compactor of size 2 contains at most one item, otherwise it would be compacted. Therefore, the bottom $H^{\prime }$ compactors have total weight

$$\sum_{h=1}^{H^{\prime }}w_{h}=\sum_{h=1}^{H^{\prime }}2^{h-1}\leq 2^{H^{\prime }}.$$

Similarly, every compactor of size $k_{h}$ contains at most $k_{h}-1=kc^{H-h}-1\leq \left(k-1\right)c^{H-h}$ items. Then, the total weight of compactors from level $H^{\prime }+1$ to *H* is:

$$\begin{array}{cc} \sum_{h=H^{\prime }+1}^{H}\;\left(k_{h}-1\right)w_{h} & \leq \sum_{h=1}^{H}\left(k-1\right)c^{H-h}2^{h-1}\\ \; & =\left(k-1\right)c^{H-1}\sum_{h=1}^{H}{\left(2/c\right)}^{h-1}\\ \; & =\left(k-1\right)c^{H-1}\frac{{\left(2/c\right)}^{H}-1}{2/c-1}\\ \; & \leq \frac{\left(k-1\right)2^{H}}{2-c}\leq \left(k-1\right)2^{H} \end{array}$$

Putting together the total weight of the bottom $H^{\prime }$ and top $H-H^{\prime }$ compactors, we obtain the upper bound on the number of items processed:

$$n\leq \left(k-1\right)2^{H}+2^{H^{\prime }}\leq \left(k-1+1/2\right)2^{H}\leq k2^{H}.$$

Plugging $n\leq k2^{H}$ into the last inequality of Equation (A1) leads to $m_{h}\leq 2{\left(2/c\right)}^{H-h}$, which is 4/c times worse than the initial derivation. Repeating the argument as in [13] and in the Section 2 of the current paper, we obtain $\sum_{h=1}^{H}\sum_{i=1}^{m_{h}}w_{h}^{2}\leq \frac{2n^{2}/k^{2}}{c^{3}\left(2c-1\right)}$. As in [13], applying Hoeffding’s inequality gives

$$P\left(|\mathrm{Err}|>\varepsilon n\right)\leq 2\exp\left(-C\varepsilon^{2}k^{2}\right)\leq \delta .$$

However, the constant *C* has changed from $2c^{2}\left(2c-1\right)$ to $C=\frac{1}{2}c^{3}\left(2c-1\right)$. Note that all asymptotic guarantees stay the same as in [13].
